# Traditional Cooking Methods Affect Color, Texture and Bioactive Nutrients of *Undaria pinnatifida*

**DOI:** 10.3390/foods11081078

**Published:** 2022-04-08

**Authors:** Shan Jiang, Meiqi Yu, Yingzhen Wang, Wei Yin, Pengfei Jiang, Bixiang Qiu, Hang Qi

**Affiliations:** 1 National Engineering Research Center of Seafood, Liaoning Provincial Aquatic Products Deep Processing Technology Research Center, School of Food Science and Technology, Dalian Polytechnic University, Dalian 116034, China; jiangshan_dlpu@163.com (S.J.); yumeiqi_dlpu@163.com (M.Y.); wangyz2036@163.com (Y.W.); dpb@gaishi.cn (P.J.); 2 Dalian Gaishi Food Co., Ltd., Dalian 116047, China; 13840940070@163.com; 3 Fujian Yida Food Co., Ltd., Fuzhou 350500, China; 13905028186@163.com

**Keywords:** *U. pinnatifida*, traditional cooking methods, quality characteristics, color, texture, bioactive nutrients

## Abstract

*Undaria pinnatifida* (*U. pinnatifida*) is an edible brown seaweed with high health value. The objective of this study was to evaluate the effect of traditional cooking methods (i.e., blanching, steaming, boiling and baking) on the color, texture and bioactive nutrients of *U. pinnatifida*, so as to screen out the traditional cooking methods more suitable for *U. pinnatifida*. In this study, methods of blanching and boiling resulted in better reduction in total color difference (0.91 ± 0.58 and 0.79 ± 0.34, respectively) and retention of chlorophyll A (62.99 ± 1.27 µg/g FW and 51.35 ± 1.69 µg/g FW), along with better elevation of fucoxanthin content (increased by 11.05% and 18.32%, respectively). Baking method got the best retention of total phenol content (1.62 ± 0.11 mg GAE/g DW), followed by methods of boiling and blanching (1.51 ± 0.07 mg GAE/g DW and 1.43 ± 0.05 mg GAE/g DW). Among these cooking methods, blanching and boiling seemed to be the more suitable for *U. pinnatifida* compared to other methods. These results could help to determine the better cooking methods for *U. pinnatifida* products and provide a scientific and theoretical basis for improving human dietary health.

## 1. Introduction

A healthy diet has been a hot topic in recent years. As the public becomes more health conscious, more and more vegetables are being considered as a part of a healthy lifestyles. Therefore, there is a growing demand for high value-added vegetables. *Undaria pinnatifida* (*U. pinnatifida*) is a large annual temperate seaweed found mainly in the coastal areas of China, Japan and South Korea [1]. *U. pinnatifida* has a high economic and medicinal value and is rich in polysaccharides, protein, vitamins, minerals, polyphenols and phytosterols [2]. The components of *U. pinnatifida* showed various biological activities, such as hypolipidemic, antihypertensive, immunoregulation and antitumor activity [3]. In addition, the application of polyphenols and pigments in seaweeds has also become a research hotspot in recent years. Utilizing their unique physicochemical properties and health functions, they could be used as preservatives, antioxidants and colorants. In a variety of foods, they could achieve the purpose of improving quality, extending storage period and developing new health foods, and have broad application prospects in food production.

In order to provide reasonable guidance for cooking food and improve the sensory and nutritional characteristics of each meal, it is necessary to determine the optimal cooking methods [4]. The color, texture, structural state and bioactive nutrients are the main factors to measure the value of vegetables, and cooking methods greatly affect these important indicators. The cooking and processing methods of vegetables can preserve/improve the nutrition and quality of vegetables to a certain extent [5]. Many studies have evaluated the effects of different cooking methods on the qualities of vegetables [6,7]. In a study on the effect of different baking conditions on the sensory quality of barley, it was found that baking in the low to medium temperature range improved the sensory quality [8]. Red cabbage cooked by the sous-vide method was more purple and more flavorful than traditionally cooked cabbage [9]. Akdaş and Bakkalbaşı [10] found that microwaving and stir-frying increased the value of the greenness in kale. Additionally, cooking methods have different effects on the bioactive ingredients in vegetables. They may increase the bioavailability of polyphenols, fucoxanthin and chlorophyll; however, it may also lead to a loss of their contents [11]. Moreover, the extent of these changes depends on a number of factors, such as the type and quality of raw materials, the used methods, the degree of heating, immersion in the processing medium, the extraction solvent, as well as the pH [12,13,14]. Giusti et al. revealed that the contents of free and bound phenolic compounds were greatly reduced during cooking and most of the phenolic compounds were leached into water [15]. Compared with sous-vide and traditional processing, cook-vide had less harm to the total phenol content of green bean. Moreover, sous-vide had better protective effect on the total phenolic content of carotenes [16]. According to the study by Gu et al., the steaming process resulted in a significant loss of phenolic/flavonoid components while the baking process had minimal impact on the active components [5].

At present, there are few studies within our knowledge comparing the effects of different traditional cooking methods on the quality and bioactive nutrients of *U. pinnatifida* and analyzing structural changes from a microscopic perspective. Therefore, in this study, four traditional cooking methods (blanching, steaming, boiling and baking) were used to compare the changes in characteristics (color, texture, moisture and microstructure) and bioactive nutrients (total phenols, chlorophyll A and fucoxanthin) of *U. pinnatifida*. The aims of this study were to: (1) compare the effects of four traditional cooking methods on the color, texture and bioactive nutrients of *U. pinnatifida*; and (2) screen for better cooking methods to preserve bioactive nutrients, texture and color in cooking. This study provides useful guidance on cooking selection of *U. pinnatifida*, as well as a theoretical basis and basic reference for cooking *U. pinnatifida* to improve human dietary health.

## 2. Materials and Methods

### 2.1. Materials and Reagents

The dried *U. pinnatifida* was supplied by Dalian LiaoHai Aquatic Food Trading Co., Ltd. (Dalian, China). It was stored in a sealing bag before analysis. Folin–Ciocalteu’s phenol reagent was obtained from Sangon Biotech (Shanghai) Co., Ltd. (Shanghai, China). Fucoxanthin (≥98%, HPLC grade), chlorophyll A (≥95%, HPLC grade) and gallic acid (≥98%, HPLC grade) were provided by Sigma-Aldrich Co., Ltd. (Shanghai, China). Methanol (HPLC grade) was purchased from Sibiquan Chemical Co., Ltd. (Shanghai, China). Acetone (HPLC grade) was supplied by Sinopharm Chemical Reagents Co., Ltd. (Shanghai, China). All other chemicals used for the analysis were analytical grade.

### 2.2. Preparation of U. pinnatifida Samples

The dried *U. pinnatifida* was immersed in deionized water until fully extended and drained to remove excess water. The rehydrated *U. pinnatifida* was then cut into equal sizes and used for subsequent experiments at 100 g per aliquot.

### 2.3. Conditions of Cooking Methods

Four traditional cooking methods were used in this study, including blanching, steaming, boiling and baking. Initial tests were carried out on each sample to optimize the cooking conditions. The shortest cooking times were used for all methods to achieve adequate palatability and taste according to the Chinese eating habits.

Blanching: 100 g of prepared *U. pinnatifida* sample was immersed in 1 L of boiling water (100 °C) for 3 min, and the blanched sample was then wiped dry and cooled on ice.

Steaming: after the water in the steamer began to boil, the *U. pinnatifida* sample (100 g) was placed in the steamer for 10 min and then quickly cooled on ice to prevent further heating.

Boiling: the *U. pinnatifida* sample (100 g) was boiled in 1 L boiling water (100 °C) for 10 min in a covered stainless-steel pot, imitating traditional cooking methods. The boiled samples were dried with kitchen papers and cooled on ice immediately.

Baking: the *U. pinnatifida* sample was baked in the oven (SCC WE 101, RATIONAL, Landsberg a. Lech, Germany) for 10 min at 160 °C.

Partially cooled samples (approximately 10 g for each sample) were used for immediate analysis of moisture, color, texture and chlorophyll A. Another portion (approximately 90 g for each sample) was placed in a drying oven (PH-070A, Shanghai YiHeng Technology Co., Ltd., Shanghai, China) at 50 °C for 16 h. The dried samples were crushed and passed through a 200-mesh sieve. The dried powder was sealed in the vacuum bag and stored at 4 °C for further analysis. The raw *U. pinnatifida* was analyzed as the control.

### 2.4. Quality Characteristics

#### 2.4.1. Color Analysis

The colors of the raw and cooked *U. pinnatifida* samples were measured using a chromameter (UltraScan Pro, HunterLab, Reston, VA, USA). In the CIE color system, the negative coordinates of a* and the positive coordinates of b* represent the green and yellow intensities, respectively [17]. The calculation of the total color difference (Δ*E*) was performed according to the formula previously described by Armesto et al. [18]:

$$\Delta E_{\mathrm{ab}}=\sqrt{{\left({L_{2}}^{*}-{L_{1}}^{*}\right)}^{2}+{\left({a_{2}}^{*}-{a_{1}}^{*}\right)}^{2}+{\left({b_{2}}^{*}-{b_{1}}^{*}\right)}^{2}}$$

where *L*_1_*, *a*_1_*, *b*_1_* are the values of the raw sample. *L*_2_*, *a*_2_*, and *b*_2_* are the values of the samples subjected to different cooking methods.

#### 2.4.2. Texture Profile Analysis

For texture profile, the *U. pinnatifida* samples were analyzed by texture analyzer (TA-XT plus, Stable Micro Systems Ltd., Vienna, UK) equipped with a 5 mm diameter (P/5) probe. The parameters were set as follows: compression variable, 30%; pre-test speed, 2.0 mm/s; test speed: 1.0 mm/s; post-test speed, 2.0 mm/s, which was according to the procedure described by Peng et al. [19].

#### 2.4.3. Low-Field Nuclear Magnetic Resonance (LF-NMR) Measurement

The water state of raw and cooked samples was determined using a LF-NMR analyzer (MesoQMR23-060H, Suzhou (Shanghai) Niumag Electronic Technology Co., Ltd., Shanghai, China). The decay signal was acquired using the Carr–Purcell–Meiboom–Gill (CPMG) pulse sequence. The main parameters were according to Jiang et al., as follows [20]: TE (time echo) = 0.5 ms, TW (time waiting) = 4000 ms, NS (number of scan) = 8, NECH (number of echo) = 8000.

#### 2.4.4. Scanning Electron Microscope (SEM)

The microstructure of raw and cooked *U. pinnatifida* was observed using a SEM (JSM-7800F, Tokyo, Japan). According to the method of Jiang et al., *U. pinnatifida* was freeze-dried in a freeze-dryer at −80 °C for 48 h. The samples obtained were fractured in liquid nitrogen and structural changes in the fracture surface were observed under SEM with ×500 magnification [20].

### 2.5. Bioactive Nutrients

#### 2.5.1. Total Phenol

The total phenolic compounds were extracted with modifications as described by Ummat et al. [21]. Briefly, *U. pinnatifida* sample powder was mixed with 50% ethanol (1:15, *w*/*v*) and sonicated for 30 min at 25 °C. The mixture was incubated in a thermostatic shaker (THZ-82, Zhibri Instruments Ltd., Changzhou, China) at 50 °C for 7.5 h at 120 rpm, then filtered through Whatman #1 filter paper (Whatman International Co., Ltd., Maidstone, UK) and the supernatant was collected. The extracts were then stored at 4 °C in the dark until analysis.

Total phenol content (TPC) was determined using the modified Folin–Ciocalteau method [20]. First 600 µL of deionized water and 50 µL of Folin–Ciocalteu phenol reagent were added sequentially to 50 µL of the extract. Next, 20% sodium carbonate solution (150 µL) was added after 1 min, then deionized water was added to the mixture to 1 mL and incubated in the dark at 25 °C for 2 h. The microplate reader (Infinite 200, Tecan Austria Co., Ltd., Grodig, Austria) was used to measure the absorbance at 760 nm. A standard curve with continuous gallic acid solution was used for calibration. TPC was expressed as gallic acid equivalent (GAE) per gram of dry weight (DW).

#### 2.5.2. Fucoxanthin

The *U. pinnatifida* sample powder was mixed with 80% methanol (1:10, *w*/*v*) and sonicated at room temperature for 30 min. The mixture was centrifuged at 3040× *g* for 10 min and the supernatant was evaporated in the rotary evaporator at 40 °C until dryness. The same volume of methanol (HPLC grade) was used to redissolve and the resulting solution was filtered through a 0.22 µm syringe filter to obtain the fucoxanthin extract.

The fucoxanthin was determined by HPLC according to the procedure described by Sui et al. [22]. Methanol was used as the mobile phase in a C18 column (5 µm, 4.6 × 250 mm, Shimadzu, Kyoto, Japan) at 40 °C with a flow rate of 0.5 mL/min. The injection volume was 10 µL, and the data acquisition time was 10 min. Detection of fucoxanthin was performed at 450 nm and the results were expressed as µg per gram of DW.

#### 2.5.3. Chlorophyll A

Chlorophyll A was extracted with modification as described by Havlíková et al. [23]. First, 5 mL acetone and 1 mL deionized water were mixed with 1 g of *U. pinnatifida* sample, sonicated at room temperature for 2 h. Then the mixture was centrifuged at 3040× *g* for 10 min and the supernatant was collected. The supernatant (5 mL) was evaporated to dryness in a rotary evaporator at 40 °C and redissolved with acetone (5 mL, HPLC grade). The solution was filtered through 0.45 µm nylon microporous filter membrane to obtain the chlorophyll A extract.

The chlorophyll A extract was also analyzed by Shimadzu HPLC series system. The C18 column (5 µm, 4.6 × 150 mm, Shimadzu, Japan) was used to achieve the separation at 30 °C. The mobile phase was 100% methanol (HPLC grade) with a flow rate of 0.8 mL/min and the total run time was 20 min. Detection was performed by a DAD at 430 nm and the injection volume was 10 µL. The results were expressed as µg per gram of fresh weight (FW).

### 2.6. Statistical Analysis

All experiments were conducted at least in triplicate. Data are reported as means ± standard deviation. Statistical comparisons were performed by analysis of variance (ANOVA) with Duncan’s multiple range tests, and the significant differences were identified at a level of *p* < 0.05 using SPSS software (ver. 20.0; SPSS Inc., Chicago, IL, USA).

## 3. Results and Discussion

### 3.1. Effects of Cooking Methods on Quality Characteristics of U. pinnatifida

#### 3.1.1. Color of *U. pinnatifida* in Different Cooking Methods

Color plays an important role in the acceptability of vegetables after cooking [24]. The effects of different cooking methods on color coordinates and total color difference (Δ*E*) were observed in Table 1. Cooking samples other than blanching significantly increased the a* value compared to the raw sample, which meant the greenness was reduced. The greenness of the blanching sample was increased, this result was consistent with the previous reported results. Pellegrini et al. evaluated the effect of different cooking methods on the color changes of three Brassica vegetables and the results showed that blanching can prevent vegetables from discoloring after they were cooked to a certain extent [25]. Additionally, the result of the reduction in greenness by steaming was consistent with the results reported by Zhong et al., who found that steaming broccoli florets lost the greenness (increased a* values) [26]. For b* value and L* value, except the b* value of the baking was significantly decreased, other cooking methods had no significant change compared with the control group. The total color difference (∆*E*) of the samples with different cooking methods was shown in Table 1, with the total color difference ranging between 0.79 ± 0.34 and 7.65 ± 0.49. The raw *U. pinnatifida* was considered as the control, baking had a greater effect on the ∆*E* than steaming (1.76 ± 0.85), blanching (0.91 ± 0.58) and boiling (0.79 ± 0.34), as the baking had a greater effect on the a* and b* values. Mashiane et al., found that steaming reduced the Δ*E* in both African pumpkin and pumpkin leaves, whilst boiling resulted in greater Δ*E* compared to raw leaves, which was contrary to our results and may be caused by differences in cooking conditions and raw materials [24].

#### 3.1.2. Effects of Cooking Methods on Texture of *U. pinnatifida*

Texture is an important sensory attribute affecting food acceptability [27]. The textural parameters of *U. pinnatifida* samples by different cooking methods were shown in Table 2, including the hardness, cohesiveness, chewiness and resilience. The results showed that the samples with different cooking methods underwent significant changes compared to the raw sample. Among them, the texture of the baking sample changed so much that the parameters could not be detected in the same way as the others. From Table 2, almost all texture parameters followed the same general trend, the degree of change caused by cooking compared to the control group was blanching < boiling < steaming, with the lowest change occurring in the blanching. Hardness, cohesiveness, chewiness and resilience were significantly reduced after various cooking methods (*p* < 0.05). This phenomenon may be due to impaired cell wall integrity, which in turn affects the textural properties [28]. Hardness was probably the most relevant texture feature for solid foods and played a key role in consumer acceptance and market value. Compared with the raw *U. pinnatifida* (1353.11 ± 50.78), the hardness values of cooked *U. pinnatifida* decreased significantly, with the steaming sample (1041.11 ± 96.87) the most obviously decreased in all samples, followed by boiling (1172.61 ± 37.69) and blanching (1182.27 ± 110.77) samples. Cohesiveness represents the internal force that holds the sample together until compression reaches the sample fracture point and depends on the properties of the sample and external factors such as humidity [29]. Chewiness referred to the energy required for chewing, which was correlated with hardness and cohesiveness [20]. Therefore, the trend of chewiness was consistent with the trends in hardness, i.e., raw (16,711.54 ± 3544.40) > blanching (10,514.54 ± 2108.38) > boiling (800.02 ± 31.39) > steaming (621.74 ± 133.73).

#### 3.1.3. LF-NMR Analysis

**Figure 1:**
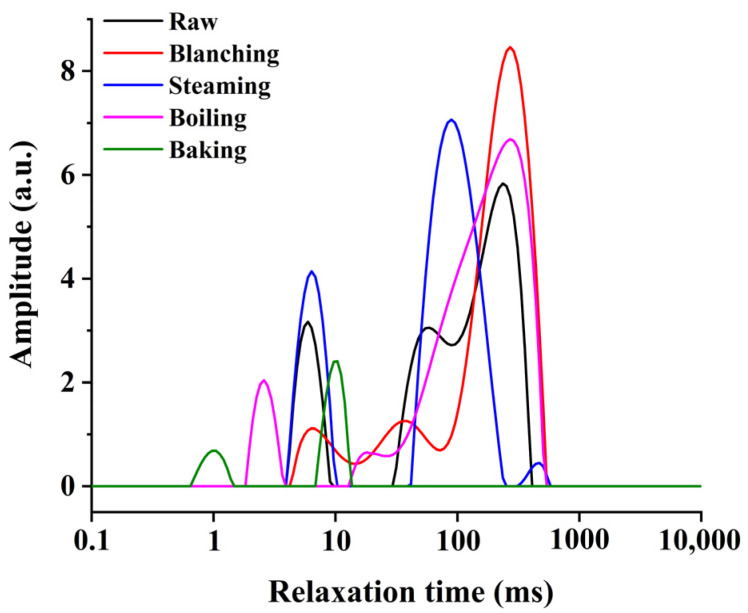
The transverse relaxation time (T_2_) curves of the raw and cooked *U. pinnatifida* under different cooking methods.

Different cooking methods can result in different moisture distribution changes, thus affecting their physicochemical properties. The transverse relaxation time of LF-NMR can reflect the water state and distribution. As shown in Figure 1, the T_2_ relaxation spectrum was obtained by multi-exponential fitting of the CPMG raw data. The smaller the T_2_, the tighter the bond between water and matter [30]. There were mainly three peaks in the T_2_ relaxation curves. The details of three peaks were T_21_ (0.1–10 ms), T_22_ (10–100 ms) and T_23_ (100–1000 ms) corresponding to the bound, immobilized and free water [31]. The results showed that the moisture changes in the *U. pinnatifida* were the most significant after baking, which showed a complete loss of free water from the baking sample and a significant reduction in T_22_ and T_21_ compared to the control group, implying a decrease in the mobility of immobilized and bound water. The fact was probably related to the higher temperature promoting the internal water losses during baking [6]. While T_23_ shifted to the right in the three cooking samples (i.e., blanching, steaming and boiling), indicating that the free water mobility of samples was stronger than raw samples. Additionally, the area of free water of the steaming sample was significantly reduced, which meant that the way of steaming caused serious loss of free water in the *U. pinnatifida.*

#### 3.1.4. Scanning Electron Microscopy (SEM)

**Figure 2:**
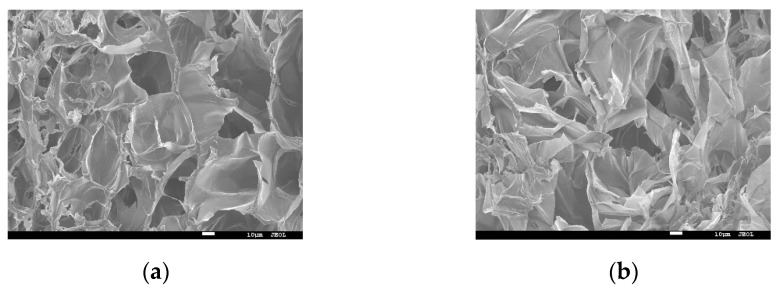

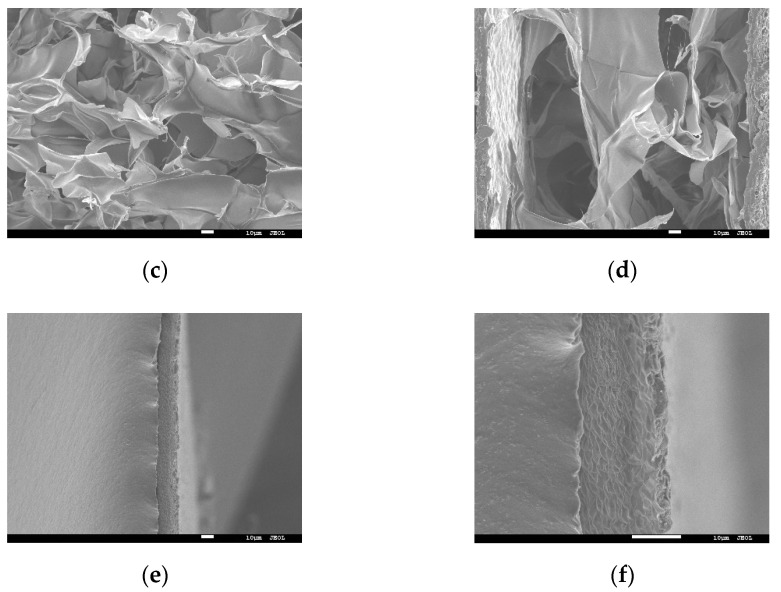
Representative scanning electron microscope (SEM) images of *U. pinnatifida* under different cooking methods (10 µm length scale bar): (**a**) raw (×500); (**b**) blanching (×500); (**c**) steaming (×500); (**d**) boiling (×500); (**e**) baking (×500); (**f**) baking (×2000).

To investigate the effect of different cooking methods on the microstructure of raw and cooked *U. pinnatifida*, freeze-dried samples were observed with scanning electron microscopy (SEM). As shown in Figure 2, the structure of the fracture surfaces of raw (Figure 2a), blanching (Figure 2b), steaming (Figure 2c), boiling (Figure 2d) and baking (Figure 2e,f) samples were shown. The microstructure of *U. pinnatifida* was significantly altered by the four different cooking methods. It could be seen that the pores of blanching samples were uneven and loose, with collapsed pore walls and disorganized arrangements, compared with raw samples. The structure of the steaming sample stuck together. After boiling, the structure of the *U. pinnatifida* changed significantly, with a significant increase in pore size and the formation of a larger cavity structure. The most pronounced changes in the samples were observed after baking treatment, where the overall sample became extremely thin and the structure was difficult to see even under multiple observations under the same conditions (×500) as the other cooking conditions, therefore needed to be observed at higher magnifications (×2000, Figure 2f). The porous structure of the baking sample was completely transformed into a uniform dense scale-like structure. This may be related to the severe loss of water from the sample during baking treatment. Yang et al. had confirmed that the structure of food changes after thermal treatment, their results showed that the surface of fried potato sticks changed under heating [32].

### 3.2. Effects of Different Cooking Methods on TPC of U. pinnatifida

The results of the evaluation of TPC in *U. pinnatifida* samples were shown in Table 3. TPC of raw *U. pinnatifida* was 1.91 ± 0.08 mg GAE/g DW, which decreased significantly after cooking (*p* < 0.05). This result indicated the breaking down of polyphenols in the *U. pinnatifida* samples during cooking. Among the four cooking methods, it was observed that the TPC in the steamed *U. pinnatifida* lost the most (32.46%), followed by the blanching samples (25.13%), boiling samples (20.94%) and baking samples (15.18%). High temperature oxidation, leaching during cooking and dissolution of phenolic compounds in hot water may lead to the loss of polyphenols during the cooking [20]. Previous studies have also reported the deleterious effects of different cooking methods on phenolic compounds in vegetables. Armesto et al. investigated some of the effects of cooking methods on the total phenol content of Galega kale [18]. It was found that all cooking methods (boiling, microwaving steaming and pressure cooking) reduced the total phenol content of kale. Sun et al. also found that the TPC in the sweet potato leaves after cooking (microwave, boiling and frying) was significantly reduced compared to untreated sweet potato leaves [33]. Lemos et al. reported that baking, steaming and boiling resulted in the loss of the TPC [34]. Jiang et al. also found a reduction in the TPC of *U. pinnatifida* after air frying and microwave cooking [20].

### 3.3. Effects of Different Cooking Methods on Fucoxanthin Content of U. pinnatifida

The fucoxanthin content was shown in Table 3. Different cooking methods had different effects on the fucoxanthin content in *U. pinnatifida*. The fucoxanthin of raw *U. pinnatifida* was 206.99 ± 7.43 µg/g DW, which increased after the four cooking treatments. Among them, the fucoxanthin content after boiling (244.91 ± 7.67 µg/g DW) and blanching (229.86 ± 2.24 µg/g DW) treatment increased significantly. The results were consistent with the study by Susanto et al., who found higher levels of fucoxanthin in brown algae Sargassum ilicifolium under different blanching treatments than in untreated ones [35]. The increase in content may be because the heat treatment may increase free fucoxanthin in the protein-bound state, such as fucoxanthin chlorophyll a/c binding protein [36]. In addition, the heat treatment may increase the diffusivity of the solvent by affecting the microstructure of the *U. pinnatifida*, thus increasing the permeability of the cell wall, which made the extraction of the fucoxanthin easier [37]. Heating inactivated fucoxanthin-destroying endogenous oxidases, such as polyphenol oxidase and peroxidase, which could prevent further fucoxanthin degradation [35].

### 3.4. Effects of Different Cooking Methods on Chlorophyll A Content of U. pinnatifida

The source of greenness in *U. pinnatifida* was mainly chlorophyll, and its content was related to a* value to a certain extent. The contents of chlorophyll A in blanching, steaming, boiling and baking samples were 62.99 ± 1.27 µg/g FW, 42.28 ± 2.13 µg/g FW, 51.35 ± 1.69 µg/g FW, and 27.04 ± 0.98 µg/g FW, respectively. These contents were significantly lower than that in the control group (83.43 ± 9.63 µg/g FW). Compared with the raw sample, the degree of chlorophyll A loss was blanching (24.50%) < boiling (38.45%) < steaming (49.32%) < baking (67.59%). The changes in chlorophyll A contents and greenness (−a*) were almost consistent. The loss of chlorophyll A content during boiling or blanching in water may be due to degradation of chlorophyll to pheophytins and cell contents were leached in boiling water [24]. Similar to our results, Mazzeo et al. observed that steamed and boiled vegetables had lower chlorophyll A content than fresh samples [4]. Possible causes were cell rupture due to heat treatment, resulting in degradation and/or loss of chlorophyll. Additionally, Gonnella et al. observed that all cooking treatments (steaming, boiling, microwaving and sous vide) significantly reduced the content of chlorophyll A of asparagus spears [38]. The most severe loss of chlorophyll A content was observed in the baking samples, probably due to the severe thermal degradation caused by the high temperature of 160 °C. Similarly, Chen et al. confirmed that the chlorophyll A content of Nori (red algae), sea lettuce (green algae) and kombu (brown algae) decreased after boiling and microwaving [39].

## 4. Conclusions

This study showed that different traditional cooking methods had different effects on the bioactive nutrients, color, texture, etc., of *U. pinnatifida*. The influence of cooking methods on bioactive nutrients is important to enhance the health benefits of cooked seaweeds for consumers. Therefore, it is of great significance to select the suitable cooking methods to reduce the loss of bioactive nutrients and quality during cooking. Based on the results of this study, blanching and boiling appeared to be better for *U. pinnatifida* than other methods. In our previous study [20], it was concluded that microwaving was more suitable for cooking *U. pinnatifida* than high temperature and pressure and air frying. However, the mildest traditional cooking methods that preserve quality characteristics and bioactive nutrients still need to be investigated in future studies.

## Figures and Tables

**Table 1:** Influence of different cooking methods on the color of *Undaria pinnatifida* (*U. pinnatifida*).

Color Properties	Raw	Blanching	Steaming	Boiling	Baking
L*	23.60 ± 0.56 ^ab^	23.48 ± 0.90 ^ab^	24.44 ± 0.98 ^a^	24.04 ± 0.37 ^ab^	23.07 ± 0.98 ^b^
a*	−4.27 ± 0.22 ^a^	−4.35 ± 0.25 ^a^	−3.52 ± 0.30 ^b^	−3.69 ± 0.19 ^b^	−1.04 ± 0.13 ^c^
b*	8.58 ± 0.52 ^a^	8.67 ± 0.57 ^a^	8.95 ± 1.26 ^a^	8.51 ± 0.21 ^a^	1.73 ± 0.47 ^b^
Δ*E*	—	0.91 ± 0.58 ^a^	1.76 ± 0.85 ^b^	0.79 ± 0.34 ^a^	7.65 ± 0.49 ^c^
Sample color	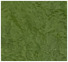	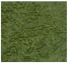	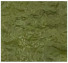	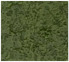	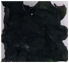

Data are expressed as mean ± standard deviation (*n* = 9). Different letters in the same row indicate significant differences at (*p* < 0.05).

**Table 2:** Influence of different cooking methods on the texture of *U. pinnatifida*.

Texture Parameters	Raw	Blanching	Steaming	Boiling	Baking
Hardness	1353.11 ± 50.78 ^a^	1182.27 ± 110.77 ^b^	1041.11 ± 96.87 ^c^	1172.61 ± 37.69 ^b^	nd
Cohesiveness	16.86 ± 2.98 ^a^	12.35 ± 1.54 ^b^	1.03 ± 0.10 ^c^	1.01 ± 0.02 ^c^	nd
Chewiness	16,711.54 ± 3544.40 ^a^	10,514.54 ± 2108.38 ^b^	621.74 ± 133.73 ^c^	800.02 ± 31.39 ^c^	nd
Resilience	0.63 ± 0.04 ^a^	0.53 ± 0.03 ^b^	0.45 ± 0.05 ^c^	0.43 ± 0.03 ^c^	nd

Data are expressed as mean ± standard deviation (*n* = 9). Different letters in the same row indicate significant differences at (*p* < 0.05). nd: not detected.

**Table 3:** Effects of different cooking methods on the contents of total phenols, fucoxanthin and chlorophyll A in *U. pinnatifida*.

Cooking Methods	Total Phenols mg GAE/g DW	Fucoxanthin µg/g DW	Chlorophyll A µg/g FW
Raw	1.91 ± 0.08 ^a^	206.99 ± 7.43 ^c^	83.43 ± 9.63 ^a^
Blanching	1.43 ± 0.05 ^c^	229.86 ± 2.24 ^ab^	62.99 ± 1.27 ^b^
Steaming	1.29 ± 0.07 ^d^	211.85 ± 2.04 ^bc^	42.28 ± 2.13 ^c^
Boiling	1.51 ± 0.07 ^bc^	244.91 ± 7.67 ^a^	51.35 ± 1.69 ^bc^
Baking	1.62 ± 0.11 ^b^	216.85 ± 12.27 ^bc^	27.04 ± 0.98 ^d^

Data are expressed as mean ± standard deviation (*n* = 3). Different letters in the same column indicate significant differences at (*p* < 0.05).

## Data Availability

Not Applicable.
